# An unusual case of adenoid cystic carcinoma presenting with isolated liver metastasis: A case report

**DOI:** 10.1097/MD.0000000000047942

**Published:** 2026-03-06

**Authors:** Ankita Simkhada, Pritha Acharya, Nisha Sharma, Bibek Shrestha

**Affiliations:** a Department of Pathology, Tribhuvan University Teaching Hospital, Kathmandu, Nepal; b Department of Internal Medicine, Tribhuvan University, Institute of Medicine, Kathmandu, Nepal.

**Keywords:** adenoid cystic carcinoma, case report, liver, metastasis, submandibular gland neoplasm

## Abstract

**Rationale:**

Adenoid cystic carcinoma (ACC) is a rare, slow-growing malignant tumor of the salivary glands characterized by perineural invasion and delayed distant metastasis. While pulmonary and osseous metastases are common, isolated hepatic metastasis as the initial presentation is exceptionally rare and diagnostically challenging.

**Patient concerns:**

A 70-year-old female presented with right hypochondrial pain and an epigastric burning sensation. She had a long-standing, painless swelling in the right submandibular region for over 10 years, for which she had not previously sought medical evaluation.

**Diagnoses:**

Imaging revealed a solitary hepatic mass measuring 11 cm × 11 cm × 9 cm involving segments IV, VII, and VIII with vascular involvement. Histopathology of the liver biopsy showed basaloid cells arranged in cribriform and tubular patterns within a myxoid stroma. Immunohistochemistry demonstrated CK7 and cluster of differentiation 117 positivity in luminal cells and SMA and p63 positivity in abluminal cells, confirming ACC. Fine needle aspiration of the submandibular swelling showed features consistent with ACC, establishing the diagnosis of metastatic ACC to the liver.

**Interventions:**

Further imaging and oncologic consultation were undertaken. Surgical resection was not feasible due to the tumor size and vascular involvement. Chemotherapy was offered.

**Outcomes:**

The patient declined further treatment after counseling regarding the metastatic nature of the disease and the potential treatment burden.

**Lessons:**

Isolated liver metastasis as the initial manifestation of ACC is exceedingly uncommon. Histopathology and immunohistochemistry are essential for diagnosis. Long-term follow-up is crucial due to the risk of delayed distant metastasis and the lack of standardized management guidelines.

## 1. Introduction

Adenoid cystic carcinoma (ACC) is a slow growing salivary gland neoplasm which constitutes <1% of all head and neck carcinomas and approximately 10% of all salivary gland neoplasms.^[1]^ It usually presents as a palpable swelling, sometimes associated with pain and numbness. Hematogenous spread to distant sites is seen in around 50% cases of ACC while lymph node metastasis is rare.^[2]^ Lung is the most common site, followed by bone, liver and brain.^[2]^

Although distant metastasis is frequent in ACC, initial presentation with isolated liver metastasis, is an extremely uncommon scenario. We report here, a case of ACC that initially presented with symptoms due to liver metastasis. Our report emphasizes the importance of awareness among clinicians and pathologists required for proper diagnosis with the aid of histopathology and immunohistochemistry, especially when a primary site of malignancy has not been established.

## 2. Case discussion

**Figure 1.**
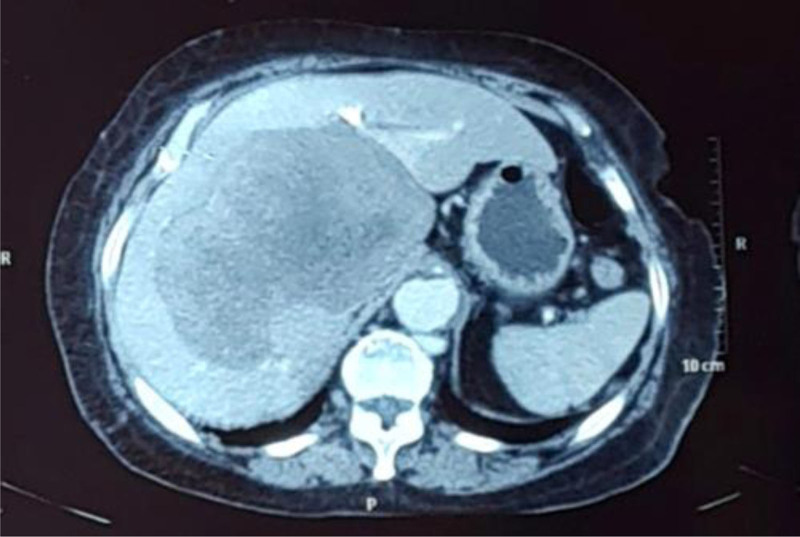
Computed tomography axial view (arterial phase) showing a large, hypodense lobulated mass involving segment IV, VII and VIII of the liver.

A 70-year-old female presented to a local health care center with complaints of right hypochondrium pain and burning sensation over the epigastrium. She was under medication for hypertension and diabetes. She was a known case of valvular heart disease. Routine blood investigations including complete blood count, liver and renal function tests were within normal limits. Ultrasonography of the abdomen showed a large mixed echoic mass in the right lobe of liver. She was then referred to our hospital for further investigation and management. Computed tomography scan of the chest and abdomen was done which showed a solitary mass of 11 cm × 11 cm × 9 cm involving segments IV, VII and VIII (Fig. 1). The mass showed no arterial enhancement however there was gradual enhancement in the delayed phase. Inferior vena cava and right hepatic vein were also involved by the mass. As the radiological features were not typical of hepatocellular carcinoma, radiological diagnosis included cholangiocarcinoma and a leiomyosarcoma arising from the inferior vena cava as the possible differentials.

**Figure 2.**
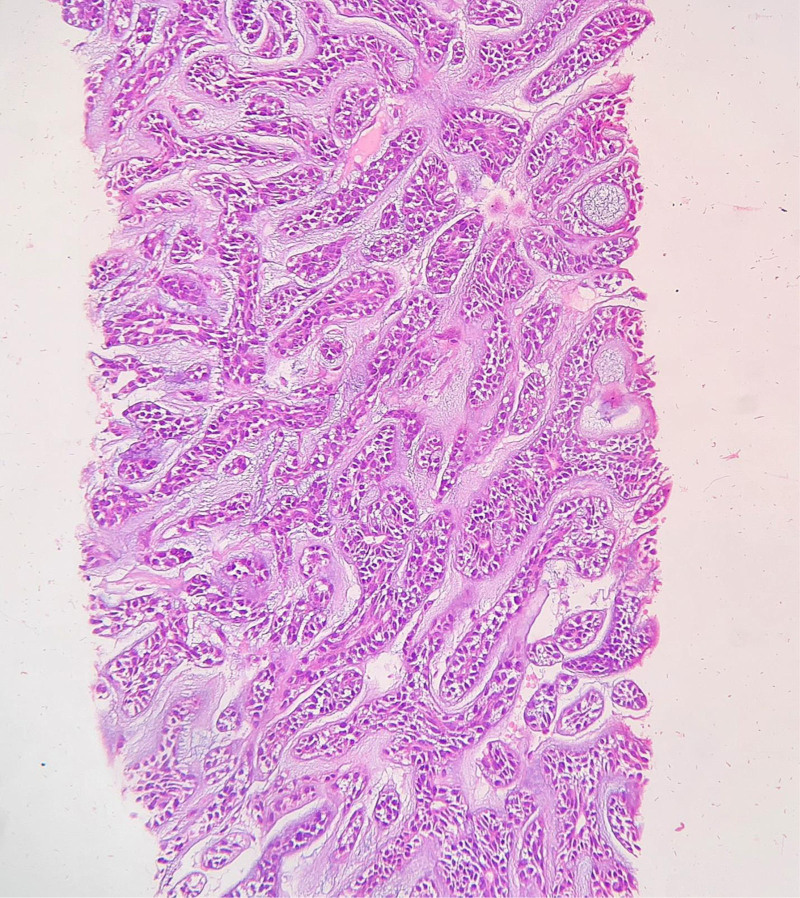
Liver biopsy showing tumor cells arranged in tubules and trabeculae. Tumor cells have scant cytoplasm and angulated nuclei. Basophilic myxoid material is also noted within the stroma (H&E, ×200).

A trucut biopy from the liver was then sent for histopathological examination. Core biopsy from the liver showed an infiltrating tumor composed of basaloid cells arranged in cords, trabeculae, cribriform pattern and occasional tubules in a conspicuous myxoid background (Fig. 2). As these features were suggestive of an epithelial tumor, the possibility of leiomyosarcoma was excluded. The histomorphology of the liver mass was reminiscent of ACC, so the patient was called for a detailed history and further examination, considering that primary ACC of liver is extremely rare.

**Figure 3.**
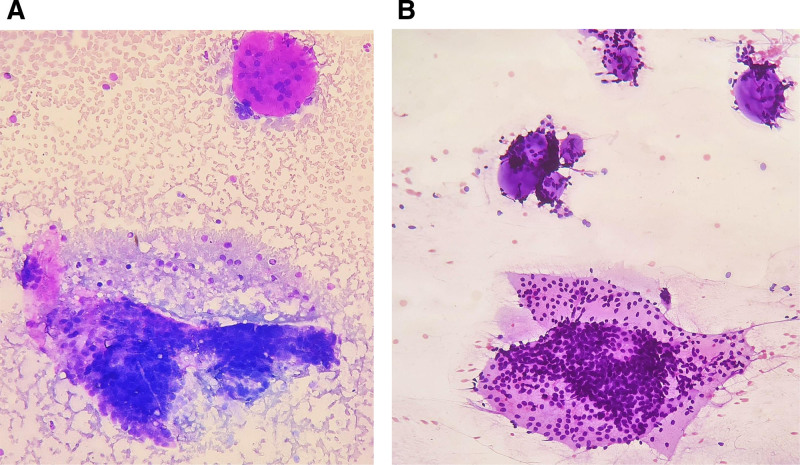
(A) Fine needle aspiration of submandibular gland mass showing sheets of basaloid cells and myxoid hyaline globule (Giemsa stain, ×200). (B) Fine needle aspiration of submandibular gland mass showing sheets of basaloid cells and rounded hyaline globules surrounded by basaloid tumor cells (H&E stain, ×200).

On examination, a firm, non-tender swelling was found over the right submandibular region measuring around 3 cm × 2 cm. On probing, she admitted that the swelling had been present for over a decade but she did not seek medical attention as it had not shown a significant increase in size over the years and was non-tender. We advised a fine needle aspiration of the swelling, to which she complied. Fine needle aspiration of the swelling showed sheets of basaloid cells along with spherical hyaline globules adherent to the tumor cells (Fig. 3A, B).

**Figure 4.**
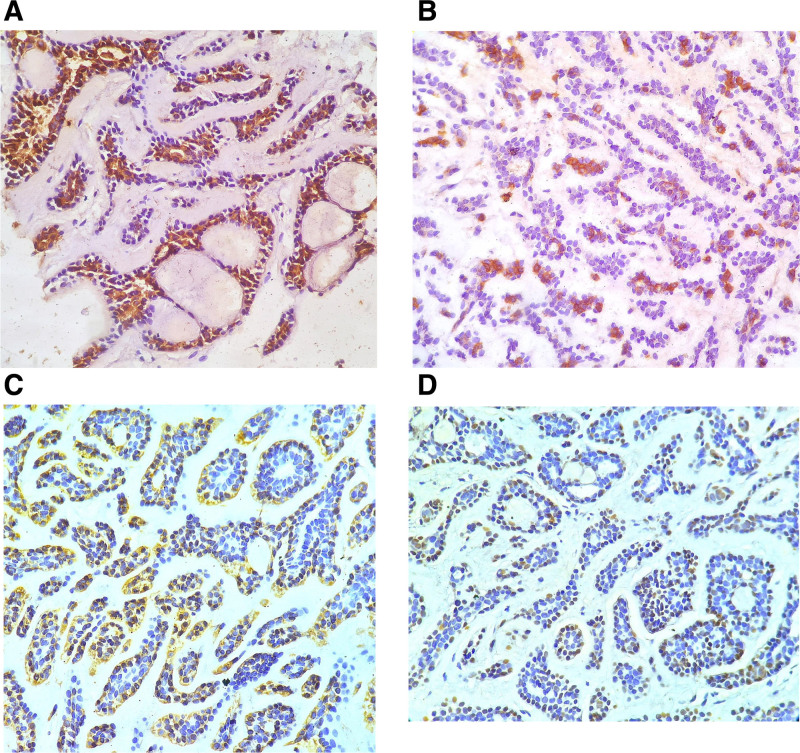
(A) Immunohistochemical stain showing cytoplasmic positivity for CK7 in ductal (luminal) cells (×400). (B) Immunohistochemical stain showing cytoplasmic positivity for CD 117 in ductal epithelial cells (×400). (C) Immunohistochemical stain showing cytoplasmic positivity for SMA in myoepithelial cells (×400). (D) Immunohistochemical stain showing nuclear positivity for p63 in myoepithelial cells (×400). CD = cluster of differentiation, SMA = smooth muscle actin.

In light of these findings, a metastatic ACC to the liver was considered and for further confirmation, immunohistochemical staining was done. CK7 and cluster of differentiation 117 showed cytoplasmic positivity in the epithelial (luminal) cells (Fig. 4A, B). Smooth muscle actin showed cytoplasmic positivity and p63 showed nuclear positivity in the myoepithelial (abluminal) cells (Fig. 4C, D). This biphasic pattern of staining confirmed a diagnosis of ACC of liver, which was considered metastatic, given the presence of submandibular gland mass.

She was then advised for MRI scan of the submandibular gland mass and referred to an oncologist to discuss further treatment options. She was informed that surgical excision of the liver mass was not possible due to its large size and involvement of vascular structures, and was offered chemotherapy. Upon knowing that her cancer had already metastasized, she was unwilling to undergo any intervention, due to concerns of pain and treatment burden at her advanced age.

## 3. Discussion

ACC is a biphasic tumor showing ductal and myoepithelial differentiation. On histopathological examination, the tumor cells are arranged in characteristic tubular, cribriform or solid architecture. Tumor cells have scant cytoplasm and hyperchromatic angulated nuclei. Basophilic matrix material is seen within the punched-out spaces in between tumor nests and lumen of tubules. The tubules are lined by inner ductal epithelial cells which show immunopositivity for CK7 and cluster of differentiation 117 and an outer layer of myoepithelial cells which are positive for smooth muscle actin and p63. These tumors are locally aggressive and perineural invasion is a common occurrence. More than 80% of the cases show a translocation (6; 9) leading to MYB–nuclear factor I/B gene fusion.^[3]^

A study by Sung et al highlights the frequent occurrence of distant metastasis seen in ACC. Their study followed 94 patients treated for ACC of which 46 showed distant metastasis. Out of the 46 cases, 30 cases had lung metastasis. Only a single case in this study showed metastasis to liver who had synchronous metastasis to the lung and bone. In addition, 25 cases showed distant metastasis in the absence of local recurrence.^[4]^ This finding has emphasized the fact that delayed distant metastasis may occur even with good locoregional control and the need for long term follow up in cases of ACC even after primary tumors have been treated adequately.

Most studies that report metastasis to liver have synchronous metastasis to other sites such as the lung and bone.^[5-7]^ Considering that the lung hosts the first capillary bed in the region, it is logical that these salivary gland neoplasm predominantly metastasize to the lungs. The soil and seed hypothesis which postulates that the “seed” represented by metastatic cancer cells can only grow in favorable “soil” which is the organ having suitable microenvironment for growth of cancerous cells may explain how metastasis occurs in other distant sites.^[8]^

Li et al have highlighted in their study, that merely 7 cases of salivary ACC were reported between 2009 and 2025 with liver metastasis as the initial presentation.^[9]^ The rarity of this presentation underscores the importance of keeping ACC in differential diagnosis of liver mass, especially when histomorphology and immunohistochemical findings are suggestive. In addition, a thorough search for previously unidentified primary sites is important as separate treatment will be warranted for primary and metastatic sites.^[9,10]^

Histological growth pattern and tumor site are variables that influence the likelihood of distant metastasis.^[4,11]^ Tumors with a solid growth pattern and those involving the major salivary are more likely to metastasize.^[4,7]^ Surgical excision with the aim of obtaining a tumor free margin is the treatment of choice for primary salivary gland tumors.^[1]^ Radiation therapy has been recommended if surgical margins are positive and in inoperable cases. Postoperative radiotherapy has shown better locoregional control and progression free survival in cases of head and neck ACC.^[12]^

In contrast to ACC in head and neck there is no consensus about the management of ACC at metastatic sites. A study by Karatzas et al highlights the combined use of multiple modalities including chemotherapy, drug eluding beads, intraoperative and percutaneous radiofrequency ablation to reduce tumor size before curative surgical resection of the liver metastasis.^[13]^

Another study by Zheng et al studied possible therapeutic modalities in cases of liver metastasis of ACC and concluded that local treatment modalities like surgery or radiofrequency ablation were more beneficial to patients with isolated liver metastasis compared to systemic therapy.^[14]^

A recent report by Zhong et al presents a unique case of metastatic ACC to the lung and liver managed with brachytherapy and stereotactic body radiotherapy for over 10 years since the diagnosis of metastatic disease. Their study reports excellent results with use of such local therapies despite the large burden of metastatic deposits with the patient tolerating these approaches with minimal adverse effects. In comparison, systemic therapy was poorly tolerated and not useful in reducing disease progression.^[5]^

In the absence of standardized treatment guidelines by organizations such as the National Comprehensive Cancer Network, management may differ between patients depending on the site, severity of metastatic disease and the availability of treatment facilities. In our case, a large tumor size, involving multiple segments of liver as well as vascular structures did not allow for surgical excision of the metastatic mass and the patient opted out of any alternative management. There are however, contrasting views regarding the role of chemotherapy for management of metastatic ACC. Some authors have raised concerns whether lack of disease progression following systemic chemotherapy is a true reflection of treatment effect or simply due to the indolent nature of the tumor.^[15]^

The overall 5 year survival rate for head and neck ACC is reported to be 68% to 90% across all grades.^[9]^ Histologic grade, tumor stage, complete excision, and distant metastasis are factors that influence overall survival.^[7]^ Studies have reported poor prognosis with shortened survival times after diagnosis of metastatic disease.^[4]^

## 4. Conclusion

To conclude, although delayed distant metastasis is common in ACC, isolated liver metastasis is an uncommon scenario that requires awareness among clinicians and pathologists alike for proper diagnosis. Long term follow up is mandatory after management of primary ACC. Management of disease at metastatic sites is challenging due to lack of specific guidelines. A larger number of systematic follow up studies on patients with distant metastasis and the outcome of various site specific treatment modalities adopted in such cases will be required to help reach a consensus on management techniques in metastatic sites.

## Author contributions

**Conceptualization:** Ankita Simkhada, Pritha Acharya.

**Writing – original draft:** Ankita Simkhada, Pritha Acharya, Nisha Sharma.

**Writing – review & editing:** Ankita Simkhada, Pritha Acharya, Nisha Sharma, Bibek Shrestha.

## References

[1] Stenman G Licitra L Said-Al-Naief N van Zante A Yarbrough W. WHO classification of head and neck tumors. In: El-Naggar AK Chan JKC Grandis JR Takata T Slootweg PJ, eds. WHO Classification of Head and Neck Tumors. 4th editio. IARC Press; 2017:164–5.

[2] Singh FM Mak SY Bonington SC. Patterns of spread of head and neck adenoid cystic carcinoma. Clin Radiol. 2015;70:644–53. 25770022 10.1016/j.crad.2015.01.013

[3] Brill LB Kanner WA Fehr A. Analysis of MYB expression and MYB-NFIB gene fusions in adenoid cystic carcinoma and other salivary neoplasms. Mod Pathol. 2011;24:1169–76. 21572406 10.1038/modpathol.2011.86

[4] Sung MW Kim KH Kim JW. Clinicopathologic predictors and impact of distant metastasis from adenoid cystic carcinoma of the head and neck. Arch Otolaryngol Head Neck Surg. 2003;129:1193–7. 14623749 10.1001/archotol.129.11.1193

[5] Zhong AY Kim SS Hopper A. Long-term treatment of metastatic adenoid cystic carcinoma with sequential brachytherapy and stereotactic body radiotherapy. Radiat Oncol J. 2024;42:237–43. 39354827 10.3857/roj.2024.00325 PMC11467482

[6] Khalid S Khan I Shafi U Atique M. A rare case of metastatic adenoid cystic carcinoma in the liver: a case report. Cureus. 2025;17:e92595. 41111729 10.7759/cureus.92595 PMC12534742

[7] Ozdemir C Karacetin D Tuna S Karadeniz A. Treatment and clinicopathologic predictors for adenoid cystic carcinomas of the head and neck. J BUON. 2011;16:123–6. 21674862

[8] Liu Q Zhang H Jiang X Qian C Liu Z Luo D. Factors involved in cancer metastasis: a better understanding to “seed and soil” hypothesis. Mol Cancer. 2017;16:176. 29197379 10.1186/s12943-017-0742-4 PMC5712107

[9] Li T Ma T Zhao Y. Submandibular adenoid cystic carcinoma presenting with liver metastasis as the initial symptom: a case report. Oncol Lett. 2025;30:1–11. 10.3892/ol.2025.15342 PMC12572763 41181629

[10] Coupland A Sewpaul A Darne A White S. Adenoid cystic carcinoma of the submandibular gland, locoregional recurrence, and a solitary liver metastasis more than 30 years since primary diagnosis. Case Rep Surg. 2014;2014:1–4. 10.1155/2014/581823 PMC4226169 25400973

[11] Zhou M Ma T Wang X. High-risk subtype: clinical manifestations and molecular characteristics of submandibular gland adenoid cystic carcinoma. Front Oncol. 2022;12:1. 10.3389/fonc.2022.1021169 PMC9800506 36591454

[12] Jensen AD Nikoghosyan AV Poulakis M. Combined intensity-modulated radiotherapy plus raster-scanned carbon ion boost for advanced adenoid cystic carcinoma of the head and neck results in superior locoregional control and overall survival. Cancer. 2015;121:3001–9. 26043145 10.1002/cncr.29443

[13] Karatzas A Katsanos K Maroulis I Kalogeropoulou C Tzorakoleftherakis E Karnabatidis D. Multi-modality curative treatment of salivary gland cancer liver metastases with drug-eluting bead chemoembolization, radiofrequency ablation, and surgical resection: a case report. J Med Case Rep. 2011;5:416. 21867491 10.1186/1752-1947-5-416 PMC3170637

[14] Zheng Y He Y Wu F Liu M Wang L Wu J. Possible local treatment for liver metastases of adenoid cystic carcinoma (ACC): single-centre experience and literature review. Transl Cancer Res. 2020;9:4573–82. 35117822 10.21037/tcr-20-1028 PMC8798837

[15] Argiris A Ghebremichael M Burtness B Axelrod RS Deconti RC Forastiere AA. A phase 2 trial of bortezomib followed by the addition of doxorubicin at progression in patients with recurrent or metastatic adenoid cystic carcinoma of the head and neck. Cancer. 2011;117:3374–82. 21246525 10.1002/cncr.25852 PMC3135694

